# Assessing the accuracy of two proxy measures for BMI in a semi-rural, low-resource setting in Guatemala

**DOI:** 10.1186/1471-2458-14-973

**Published:** 2014-09-19

**Authors:** Jonathan N Maupin, Daniel J Hruschka

**Affiliations:** School of Human Evolution and Social Change, Arizona State University, PO Box 872402, Tempe, AZ 85287-2402 USA

**Keywords:** Body mass index, Self-report, Obesity, Silhouettes, Low-income country

## Abstract

**Background:**

Validation studies of self-reported BMI are limited to populations in high-income countries or urban settings. Here, we assess the accuracy of two proxy measures of measured height, weight and BMI – self-reported values and the Stunkard figure scale – in a semi-rural population in Guatemala.

**Methods:**

Self-reported values and Stunkard figure selection were elicited prior to biometric measurements from a total of 175 non-pregnant women recruited based on a stratified random sample of households, with 92 women providing full data for validation across measures.

**Results:**

86.3% of participants self-reported weight and 62.3% height. Among those responding, self-reported weight is highly accurate though lower relationships for height contribute to error in reported BMI. The Stunkard scale has a higher response rate (97.1%) and while less accurate in predicting BMI values, more accurately predicts BMI categories.

**Conclusions:**

Self-reported measures are more accurate than the Stunkard scale in estimating BMI values, while the latter is more accurate in estimating BMI categories. High non-response rates and lower correlations between reported and measured height caution against using self-reported biometric data other than raw weight in low-resource settings.

**Electronic supplementary material:**

The online version of this article (doi:10.1186/1471-2458-14-973) contains supplementary material, which is available to authorized users.

## Background

Self-reported height and weight are commonly used to calculate BMI and to estimate the prevalence of overweight and obesity among populations. Inexpensive, non-intrusive, and quick to administer, self-reported values are an attractive option for sampling large populations or in contexts where biometric measurements are unavailable. While several studies raise concerns about the accuracy of self-report due to significant mismatches between self-reported and measured values, a large number of studies from educated populations in industrialized nations find that self-reported measurements are highly correlated with measured biometric data [1–3]. Paradoxically, there are few, if any, such validation studies in low-income, semi-literate, rural settings where it may be most difficult to obtain direct measurements of height and weight. The accuracy, or even possibility, of self-reported values among these populations may vary greatly due to issues of literacy, education, levels of food insecurity, time since last biometric measurements, and social desirability which have all been shown to influence error in self-reports [4–7]. As such, there is a need to evaluate the accuracy of self-reported biometric data among populations in rural, low-income, semi-literate settings.

In the place of self-reports of height and weight, a growing number of researchers have also examined the use of alternative measures to estimate body size, particularly the Stunkard figure rating scale [8]. The Stunkard scale consists of nine gender-specific body figures increasing in size from skinny [1] to obese [9], from which individuals are asked to identify the figure that best represents their current body size. The scale is easy to administer and has been validated in several international contexts as a measure of perceived body size as well as body image dissatisfaction [9]. Linking measured BMI values to self-selected Stunkard body figures, researchers have identified the optimal body figure cut-off points for identifying underweight, overweight, and obese individuals in various populations as well as estimating the BMI values for each of the nine gender-specific body figures [10–13]. While there is some overlap in the optimal cut-off points for identifying overweight and obese individuals across studies, the potential application of specific cut-offs for Stunkard figures across populations is uncertain given the possibility of cross-cultural or population differences in self-assessment and identification of body size.

In this paper, we assess the accuracy of self-reported height, weight, and BMI against measured values as well as the effectiveness of the Stunkard figure scale for classifying individuals as overweight and obese among a group of Maya and non-Maya in a semi-rural community in the Central Highlands of Guatemala. The accuracy of either method has not been validated in low-income countries, and particularly semi-rural communities, and thus this study provides a valuable assessment of these two methods for estimating height, weight, or BMI in a context where they may be highly valuable for researchers and public health officials.

## Methods

This study was conducted in a semi-rural community in the Central Highlands of Guatemala with a population of approximately 6,000 in the town center. Participants were recruited based on a stratified random sample of households in the community. Prior to interviews, researchers counted and mapped each household in the community, designating them by neighborhood. Households were numbered for each neighborhood and selected based on a random number generator. To have a representative sample, we randomly sampled households from neighborhood clusters, with the number of households sampled from each neighborhood proportional to the total number of households in the neighborhood. Researchers visited each randomly selected household twice to recruit participants. If no one responded after two visits, researchers moved to the next household on the randomly generated list. Researchers requested interviews from both the male and female head of households, although few men were available for interviews as the majority work outside the home during the day. If the heads of household were not present, individuals over 18 years of age were recruited to participate. In total, we counted 941 households in the community. We sampled a total of 287 households (30.5%). 80 households declined to participate (27.9% of households recruited). In total, we completed interviews in 206 households, which constitute 22% of all households in the community. We interviewed 185 women in 176 houses. In nine households we interviewed two women. The remaining households and interviews represent interviews conducted with men, which we do not present in this paper.

Human Subjects oversight for this research was provided by the Office for Research Integrity & Assurance at Arizona State University (protocol #1205007796). All individuals provided written consent to participate in the survey as well as to have their height and weight measured prior to initiating the interview.

Of the 185 women interviewed, a total of 175 non-pregnant women allowed their height/weight to be measured. Of these, 151 (86.3%) provided an estimate of their weight, 109 (62.3%) an estimate of height, and 170 (97.1%) a Stunkard assessment for a total of 92 (52.3%) women with the necessary BMI estimates for validation (see Table 1 for participant demographics).

**Table 1:** **Participant demographics and BMI categories**

	n	%
Age		
18-24	9	9.8
25-29	15	16.3
30-34	15	16.3
35-39	15	16.3
40-44	11	12
45-49	8	8.7
50-54	9	9.8
55-59	6	6.5
60-64	3	3.3
65≤	1	1.1
Ethnicity		
Maya	33	35.9
Ladino	57	62
Not reported	2	2.2
Education		
≤6th grade	33	35.9
>6th grade	55	59.8
Not reported	4	4.3
Last time weighed		
≤1 year	64	69.6
>1 year	28	30.4
Measured BMI category		
Below average	1	1.1
Average	35	38.0
Overweight	34	37.0
Obese	22	23.9

Participants were first asked to estimate their current height and weight. Weight was reported in pounds (lbs) and height in meters (m). While Guatemala uses the metric system for length, body weight is measured in American pounds (0.454 kilograms). As such, we use pounds rather than kilograms in this paper in order to stay consistent with participants’ reports. Body image perception was elicited with the Stunkard body figure scale. Participants were asked which figure, out of nine gender-specific body images increasing in size from skinny [1] to obese [9], most accurately reflects their current body size.

Upon completion of the interview, participants’ heights and weights were measured to within the nearest 0.1 cm and 0.1 kg, respectively, with a stadiometer and digital scale, following protocols established by INCAP for Guatemala [14]. Each measurement was taken twice with participants’ shoes and any outerwear (jacket/sweater) removed. If there was more than a 0.05 kg or 0.5 cm difference in the results, the measurement was taken a third time and the average of the two closest values was utilized. We subtracted 1.5 kg from the resulting weight to adjust for clothing, per INCAP’s protocols [14].

We examined the following possible factors affecting non-reporting of height and weight as well as accuracy of self-reported rates. These included: age (categorized into age groups), ethnicity (Maya/non-Maya), education (dichotomized as over/under 6th grade), self-reported literacy (able to read/write in Spanish) and time since last weighing (dichotomized as less than/more than 1 year ago). The dichotomization of education into over/under 6th grade is a standard practice for studies in Guatemala [15].

BMI (kg/m^2^) was calculated for both measured and self-reported values. The difference between measured and self-reported values (error) was calculated by subtracting self-reported from the measured values for height, weight, and BMI. BMI categories were calculated based upon international standard cut-offs of <18.5 for underweight, <25 for average, <30 for overweight, and ≥30 for obesity [16].

Descriptive statistics and 95% confidence intervals were calculated to examine the mean values. To assess factors influencing non-reporting of height and weight, we fit a logistic regression predicting reporting of height and weight by age, ethnicity, education, literacy, and time since last weighing. Spearman correlations were used to estimate the relationships between ordinal variables. One-way ANOVAs were used to test for significant differences in mean and absolute mean errors between measured and self-reported values based on specific characteristics. Linear regressions were calculated to identify predictors of both BMI as well as errors in height, weight, and BMI. To estimate the accuracy of the Stunkard figures in categorizing overweight and obese individuals, we calculated the specificity and sensitivity for overweight and obesity and utilized receiver operating characteristic (ROC) curves. Cross-tabulations were analyzed to compare categorizations across measured, self-reported, and body image BMI values.

## Results

### Measured and self-reported height and weight

Examining the patterns of non-response rates for self-reported values among the 175 non-pregnant women interviewed, we find that, when controlling for age, ethnicity, literacy, and time since last weighing, more highly educated women have greater odds (8.2, CI 2.45-27.23, p < 0.005) of reporting their height. In regards to weight, however, we find no significant factors associated with the rate of self-report, although there is a trend that women weighed within the last year have a greater odds of self-reporting their weight 3.34 (CI 0.96-11.65, p = 0.058).

Table 2 presents the averages for measured and self-reported height and weights among the 92 women who provided all necessary data. There is a strong correlation between measured and self-reported weight (r = 0.884, p < 0.000), but lower correlations between measured and self-reported height (r = 0.788, p < 0.000). The correlation between measured and reported BMI is 0.783 (p < 0.000). Self-reported BMI explains 63.4% of the variance in measured BMI.

**Table 2:** **Mean measured and self-reported biometrics with average error (SD)**

	Measured	Self-reported	Difference
Height (m)	1.51 (±0.066)	1.53 (±0.0869)	-0.027 (±0.056)
Weight (lbs)	135.19 (±24.212)	134.97 (±23.76)	0.227 (±10.107)
BMI	27.06 (±4.61)	26.17 (±4.649)	0.891 (±2.957)

Participants (the 92 women who provided all data) on average underestimated their weight by 0.227 lbs (±10.1) and overestimated their height by 2.7 cm (±5.61), with an average underestimation of BMI by 0.891 (±2.96). The average absolute value of error for women is 6.48 lbs (±7.73) for weight, 4.3 cm (±4.5) for height, and 2.19 (±2.16) for BMI. For height (t(91) = -4.564, p < 0.000) and BMI (t(91) = 2.89, p < 0.05) there are significant differences between measured and self-reported values.

Cross-tabulation analysis indicates a moderate relationship between measured and self-reported BMI category (Kappa = 0.416, p < 0.000), and 39.1% of women were misclassified in BMI category based upon self-reported values.

Using one-way ANOVAs to analyze variation in signed error, we find that overweight/obese women underestimate their weight more than average/underweight women (F = 4.497, p < 0.05), while there is a trend that non-Maya women overestimate their height more than Maya women (F = 3.086, p = 0.051). For BMI, individuals weighed more than one year ago significantly underreport their BMI (F = 6.509, p < 0.05). Additionally, obese women significantly underreport their BMI compared to average/underweight women (F = 3.303, p < 0.05). In the Additional file 1: we provide graphs to detail the differences in error among women by BMI category.

Linear regressions on the signed error for weight, height, and BMI with the variables of age, education (high/low), ethnicity (Maya/non-Maya), measured BMI, the time last weighed (ranging from 1 month to over 5 years), as well as whether the respondents’ reported height or weight ended in either a “0” or “5” (end-digit bias). For weight, the only significant predictor is measured BMI (B = 0.492, p < 0.05) as higher BMI predicts greater underreporting of weight. For height, we find that education (B = -0.032, p < 0.05), ethnicity (B = -0.016, p < 0.005), and the last time weight (B = -0.012, p < 0.05) predict the signed error as higher educated and Ladino individuals overestimate their height more than lower-educated and Maya individuals. Additionally, increases in the time since last weighing also predict over-reporting height. For BMI signed error, we find that ethnicity (B = 0.163, p < 0.05), measured BMI (B = 0.163, p < 0.05), and time since last weighing (B = 0.419, p < 0.05) are significant, as increasing BMI and time since last weighing predict greater underreporting of BMI, while Ladinos underreport BMI more than Maya individuals.

For the absolute difference between measured and reported values, we find no significant variation in error for height and weight based on ethnicity, education, time since last weighing, or BMI category. For BMI, we find that higher educated women have lower absolute error in self-reported BMI estimates (F = 4.20, p < 0.05).

We also conducted linear regressions for the absolute error for each of the measures with the variables of age, education (high/low), ethnicity (Maya/non-Maya), measured BMI, the time last weighed (ranging from 1 month to over 5 years), and the end-digit bias for height or weight. We find no relationship between the variables and the absolute error between measured and self-reported values for weight and BMI. For height, we find that both ethnicity (B = 0.009, p < 0.05) and time since last weighing (B = 0.008, p < 0.05) predict the absolute error (R^2^ = 0.123) as Ladina women and those who were weighed more recently showing less error.

### Measured and self-reported bmi and contour figure scales

The average body figure selected on the Stunkard figure scale was 4.5 (±1.64). Table 3 presents the participant distribution and average BMI for each silhouette selected. The Spearman correlation between measured BMI and silhouette number is 0.727 (p < 0.000). Following Kaufer-Horwitz, Martinez [10], we used simple linear regression to predict BMI based on silhouette selection. The model is statistically significant (p < 0.000), explaining 48% of the variance, and allowing us to calculate BMI values for each figure based on the following equations:

**Table 3:** **Measured and predicted BMI for Stunkard Silhouette numbers**

Stunkard body figure	n	%	Measured BMI			Predicted BMI		
			Lower 95% CI	Mean	Upper 95% CI	Lower 95% CI	Mean	Upper 95% CI
1	5	5.4	21.173	22.823	30.736	19.823	20.248	20.672
2	4	4.3	20.706	21.388	25.870	21.344	22.194	23.042
3	12	13.0	21.241	23.696	29.580	22.865	24.14	25.412
4	27	29.3	22.526	25.636	31.048	24.386	26.086	27.782
5	23	25.0	25.986	28.042	31.804	25.907	28.032	30.152
6	8	8.7	26.980	29.830	38.606	27.428	29.978	32.522
7	11	12.0	30.309	32.501	38.009	28.949	31.924	34.892
8	1	1.1	-	35.262	-	30.47	33.87	37.262
9	1	1.1	-	36.645	-	31.991	35.816	39.632



where x is the silhouette number and y is BMI. The standard error for the regression slope is 0.214. The beta value for the Stunkard silhouette in our model is lower than Kaufert et al.’s model (2.435). Based on the average BMI results (see Table 3), overweight ranged from Figures four to six, while obesity ranged from Figures seven to nine. However, the 95% confidence intervals for each figure are large, and BMI values begin to overlap beginning with figures 2/3, limiting the ability to estimate BMI values based upon the silhouette numbers. The number of participants who selected figures at the extremes of the Stunkard scale (e.g. 1–2 or 8–9) is small, which may distort the results of the regression model. However, visual plots of the measured and predicted BMI (included in the Additional file 1) show a strong linear pattern with higher CI at the extremes.

**Figure 1:**
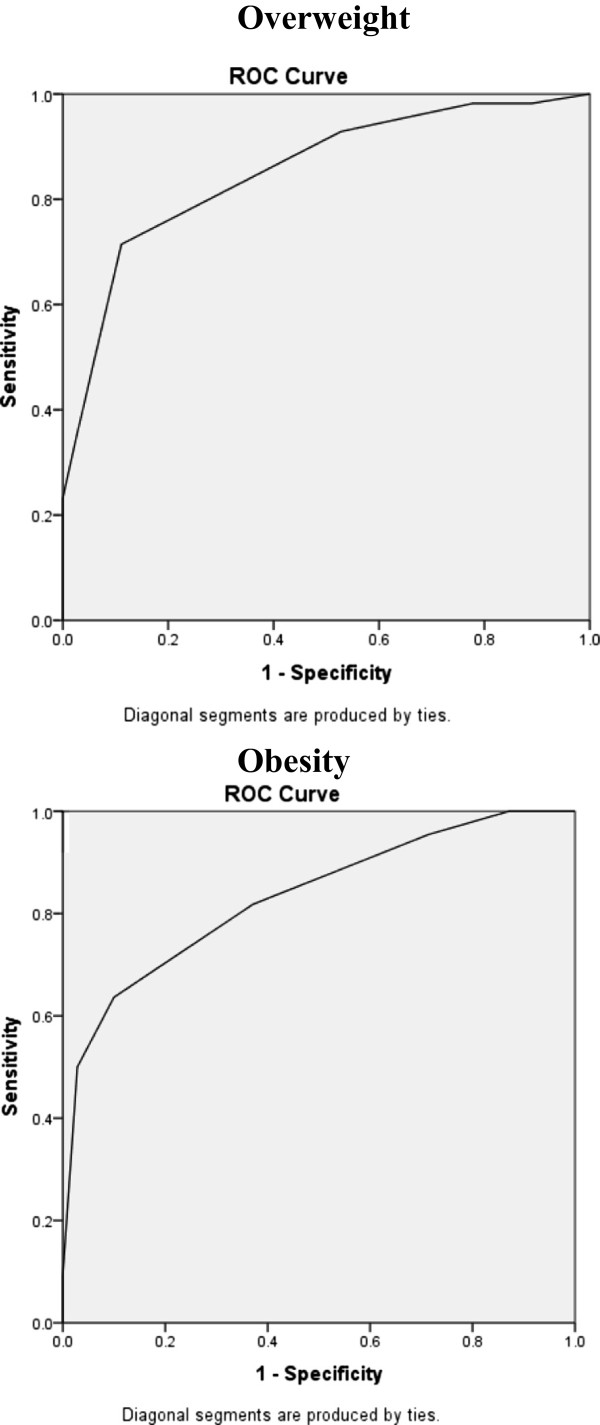
**Receiver operating characteristic (ROC) curves for overweight and obesity.**

ROC curves were calculated to assess the accuracy of classifying women as both overweight and obese based upon their silhouette selection (Figure 1). The accuracy of classification of overweight individuals was high (AUC = 0.853, CI 0.776-0.931, p < 0.000). The optimal cut-off for overweight, maximizing sensitivity and specificity, is body Figure five. For obesity, the ROC was significant (AUC = 0.832, CI 0.728-0.937, p < 0.000), and the optimal cut-off point was body Figure six, although the small number of participants who selected body sized above 7 cautions against the reliability of this cut-off.

Utilizing these cut-offs for average/underweight (<5), overweight [5], and obese [6–9], we conducted cross-tabulation analyses to analyze the reliability of categorizing individuals based on the Stunkard scales compared to measured BMI category Kappa values are moderate, 0.485 (p < 0.00), and 32.6% of women were misclassified utilizing the BMI categories derived from the Stunkard scale.

## Discussion and conclusions

In this study, we assessed the accuracy of two proxies for measured BMI—calculations from self-reported height and weight and the Stunkard body figure scale—in a low-income setting in semi-rural Guatemala. We find moderate to high rates of non-response to self-reported anthropometrics, with 86.3% of all non-pregnant women interviewed reporting weight and 62.3% reporting height, leaving 52.6% (92 participants) reporting measurements sufficient to calculate BMI. Although weight estimates were generally strongly correlated with actual measurements (r = 0.884), height estimates showed a lower correlation with actual measurements (r = 0.788), leading to a correlation between self-reported and measured BMI of r = 0.783. Nearly all women interviewed (97.1%) reported their body size on a Stunkard scale. However, among the 92 women included in the study, these estimates correlated slightly less closely with measured BMI (r = 0.727) than with self-reported BMI. Higher educated women have greater odds of reporting their height, and there is a trend that women weighed within the last year are more likely to self-report weight.

The rate of non-response is rarely (if ever) provided in studies assessing the accuracy of self-reported biometric data, and is not included in calls for standardized reporting of statistics and procedures [1, 17]. Yet, identifying the rate of non-response, and associated factors, is essential to estimating the proportion of the population that may be excluded in studies utilizing self-reports. While the issue of non-response may be less of a concern among educated populations in high- and middle-income countries where the majority of these studies are conducted, it may be a significantly limiting factor in low-income countries or semi-literate populations with less frequent biometric measurements.

The patterns of error in height, weight, and BMI roughly reflect general trends identified in the literature of underestimating weight and overestimating height. However women’s underestimation of weight by 0.227 lbs is much lower than Gorber et al.’s [1] review of 64 studies, Moreover, women’s overestimation of height by 2.7 cm is at the higher end of this range.

The Stunkard scale has the advantage of a higher response rate (97.1%), but has a much lower accuracy relative to self-reported BMI (r = 0.56). These correlations are slightly lower than those reported in other research comparing BMI with Stunkard silhouettes, which are generally >0.7 for both men and women. While there is too much overlap in confidence intervals to predict BMI values based on silhouette selection, and the limited number of women selecting figure sizes over 7, the ROC curves do identify optimal cut-off points for classifying individuals as overweight (Figure five), and obese for women (Figure six). These cut-offs replicate other estimates from diverse populations, including Bulik et al.’s [11] study among Caucasians in Virginia and Kaufer-Horwitz et al.’s [10] research in Mexico City, who both identify Figure six as the optimal cut-off for obesity. Additionally, our cut-off for overweight (Figure five) matches that identified by Lo et al.’s [13] research among Chinese adolescents. These studies all find high AUC (>0.8), suggesting that the Stunkard scales are effective in classifying individuals as average/under, overweight, and obese. Indeed, the rate of misclassification for BMI categories is lower for the Stunkard scale (32.6%) than self-reported biometric data (39.1%). Thus, while the Stunkard scale is less accurate than self-reported values for estimating BMI values, it is more reliable for classifying women as overweight.

In summary, among individuals in a semi-rural community in Guatemala, self-reported values for height and weight are more accurate than the Stunkard scale for estimating BMI values, while the Stunkard scale is more reliable for classifying individuals as overweight. Self-reported values have significant limitations, however. While reported weight correlates strongly with measured values for women, the correlation between reported and measured height is lower and contributes to error in estimating BMI. Moreover, the rates of non-response, particularly for height, limit the potential use of self-reported BMI in among populations with low education, illiteracy, and lack of access to or utilization of health services for periodic measurements. Paradoxically, it is these contexts where the use of self-reported measures may be most appealing to researchers. The lack of reporting non-response patterns and the focus on educated populations in high- and middle-income countries limits our ability to determine if these issues are unique to this particular, and albeit limited, sample. More validation studies in semi- and rural areas of lower-income countries are needed in order to determine the feasibility and accuracy of proxy measures for estimating BMI.

## Electronic supplementary material

Additional file 1: Figure S1: Mean Absolute Error in Self-Reported BMI by BMI category. **Figure S2.** Mean Absolute Error in Self-Reported Height by BMI category. **Figure S3.** Mean Absolute Error in Self-Reported Weight by BMI category. **Figure S4.** Mean Measured BMI for Stunkard Figure Sizes. Note: There are no 95% CI bars for Figures eight and nine as only one individual selected the respective body figure number, respectively. **Figure S5.** Mean Predicted BMI for Stunkard Figure Sizes. (DOCX 51 KB)

## References

[1] Gorber SC, Tremblay M, Moher D, Gorber B (2007). Diagnostic in obesity comorbidities - A comparison of direct vs. self-report measures for assessing height, weight and body mass index: a systematic review. Obes Rev.

[2] Engstrom JL, Paterson SA, Doherty A, Trabulsi M, Speer KL (2003). Accuracy of self-reported height and weight in women: an integrative review of the literature. J Midwifery Womens Health.

[3] Bowman RL, Delucia JL (1992). Accuracy of self-reported weight - a metaanalysis. Behav Ther.

[4] Lyons A-A, Park J, Nelson CH (2008). Food insecurity and obesity: a comparison of self-reported and measured height and weight. Am J Public Health.

[5] Niedhammer I, Bugel I, Bonenfant S, Goldberg M, Leclerc A (2000). Validity of self-reported weight and height in the French GAZEL cohort. Int J Obes Relat Metab Disord.

[6] Elgar FJ, Roberts C, Tudor-Smith C, Moore L (2005). Validity of self-reported height and weight and predictors of bias in adolescents. J Adolesc Health.

[7] Villanueva EV (2001). The validity of self-reported weight in US adults: a population based cross-sectional study. BMC Public Health.

[8] Stunkard AJ, Sorensen T, Schulsinger F (1983). Use of the Danish Adoption Register for the study of obesity and thinness. Res Publ Assoc Res Nerv Ment Dis.

[9] Brewis A (2011). Obesity: Cultural and Biocultural Perspectives.

[10] Kaufer-Horwitz M, Martinez J, Goti-Rodriguez LM, Avila-Rosas H (2006). Association between measured BMI and self-perceived body size in Mexican adults. Ann Hum Biol.

[11] Bulik CM, Wade TD, Heath AC, Martin NG, Stunkard AJ, Eaves LJ (2001). Relating body mass index to figural stimuli: population-based normative data for Caucasians. Int J Obes Relat Metab Disord.

[12] Fitzgibbon ML, Blackman LR, Avellone ME (2000). The relationship between body image discrepancy and body mass index across ethnic groups. Obes Res.

[13] Lo W-S, Ho S-Y, Mak K-K, Lam T-H (2012). The Use of Stunkard’s figure rating scale to identify underweight and overweight in Chinese adolescents. PLoS One.

[14] PAHO (2007). Central American Diabetes Initiative (CAMDI): Survey of Diabetes, Hypertension, and Chronic Disease Risk Factors. Villa Nueva, Guatemala, 2006.

[15] Chaparro C (2012). Household Food Insecurity and Nutritional Status of Women of Reproductive Age and Children under 5 Years of Age in Five Departments of the Western Highlands of Guatemala.

[16] WHO (1998). Obesity: Preventing and Managing the Global Epidemic: Report of a WHO Consultation on Obesity.

[17] Engstrom JL (1988). Assessment of the reliability of physical measures. Res Nurs Health.

[CR18] The pre-publication history for this paper can be accessed here: http://www.biomedcentral.com/1471-2458/14/973/prepub

