# Supplementary material for: Assessing the accuracy of two proxy measures for BMI in a semi-rural, low-resource setting in Guatemala
Source: BMC Public Health. 2014 Sep 19;14:973. doi: 10.1186/1471-2458-14-973 (PMC4190281; doi:10.1186/1471-2458-14-973)
Supplement: Supplementary file 1 — Additional file 1: Figure S1: Mean Absolute Error in Self-Reported BMI by BMI category. Figure S2. Mean Absolute Error in Self-Reported Height by BMI category. Figure S3. Mean Absolute Error in Self-Reported Weight by BMI category. Figure S4. Mean Measured BMI for Stunkard Figure Sizes. Note: There are no 95% CI bars for Figures eight and nine as only one individual selected the respective body figure number, respectively. Figure S5. Mean Predicted BMI for Stunkard Figure Sizes. (DOCX 51 KB) [file 12889_2013_7099_MOESM1_ESM.docx]

**Title: Assessing the Accuracy of Two Proxy Measures for BMI in a Semi-rural, Low-Resource Setting in Guatemala.**

Jonathan N. Maupin and Daniel Hruschka

Additional file 1.


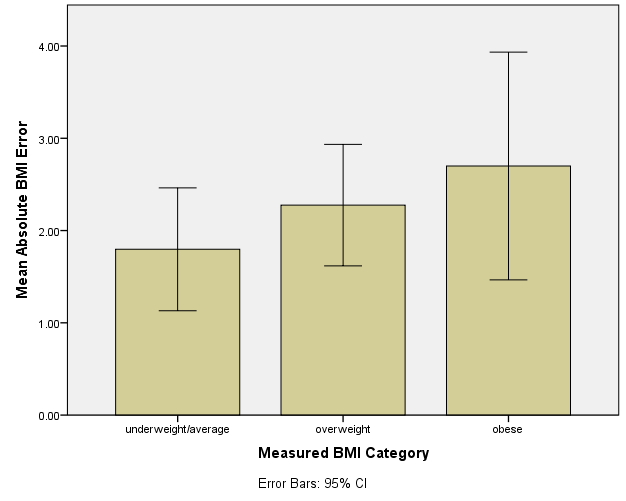


Figure S1. Mean Absolute Error in Self-Reported BMI by BMI category.


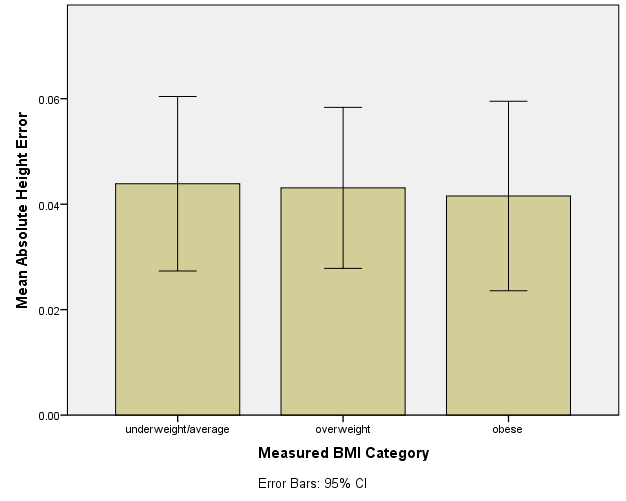


Figure S2. Mean Absolute Error in Self-Reported Height by BMI category.


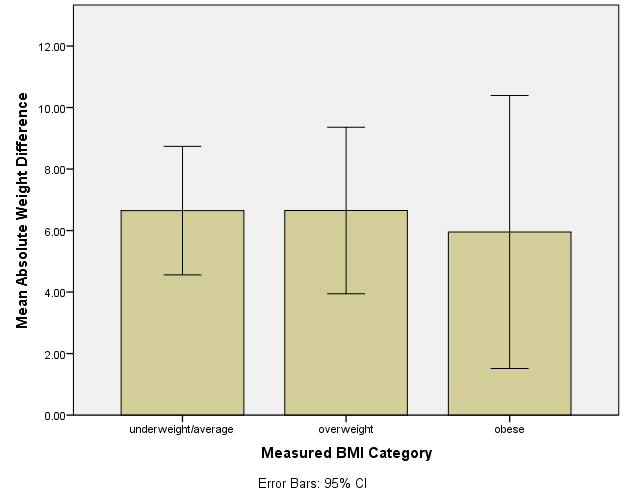


Figure S3. Mean Absolute Error in Self-Reported Weight by BMI category.

Figure S4. Mean Measured BMI for Stunkard Figure Sizes

Note: There are no 95% CI bars for Figures 8 and 9 as only one individual selected the respective body figure number, respectively.

Figure S5. Mean Predicted BMI for Stunkard Figure Sizes
